# The Role of Socio-Demographic Factors in the Coverage of Breast Cancer Screening: Insights From a Quantile Regression Analysis

**DOI:** 10.3389/fpubh.2021.648278

**Published:** 2021-04-15

**Authors:** Lilu Ding, Svetlana Jidkova, Marcel J. W. Greuter, Koen Van Herck, Mathieu Goossens, Harlinde De Schutter, Patrick Martens, Guido Van Hal, Geertruida H. de Bock

**Affiliations:** ^1^Department of Epidemiology, University Medical Center Groningen, University of Groningen, Groningen, Netherlands; ^2^Department of Social Epidemiology and Health Policy, University of Antwerp, Antwerp, Belgium; ^3^Department of Public Health and Primary Care, Ghent University, Ghent, Belgium; ^4^Center for Cancer Detection, Flanders, Belgium; ^5^Department of Radiology, University Medical Center Groningen, University of Groningen, Groningen, Netherlands; ^6^Department of Robotics and Mechatronics, University of Twente, Enschede, Netherlands; ^7^Research Department, Belgian Cancer Registry, Brussels, Belgium

**Keywords:** breast cancer, mammography screening, coverage, social inequality, determinant, quantile regression

## Abstract

**Background:** In Flanders, breast cancer (BC) screening is performed in a population-based breast cancer screening program (BCSP), as well as in an opportunistic setting. Women with different socio-demographic characteristics are not equally covered by BC screening.

**Objective:** To evaluate the role of socio-demographic characteristics on the lowest 10th and highest 90th quantile levels of BC screening coverage.

**Methods:** The 2017 neighborhood-level coverage rates of 8,690 neighborhoods with women aged 50–69 and eligible for BCSP and opportunistic screening were linked to socio-demographic data. The association between socio-demographic characteristics and the coverage rates of BCSP and opportunistic screening was evaluated per quantile of coverage using multivariable quantile regression models, with specific attention to the lowest 10th and highest 90th quantiles.

**Results:** The median coverage in the BCSP was 50%, 33.5% in the 10th quantile, and 64.5% in the 90th quantile. The median coverage of the opportunistic screening was 12, 4.2, and 24.8% in the 10th and 90th quantile, respectively. A lower coverage of BCSP was found in neighborhoods with more foreign residents and larger average household size, which were considered indicators for a lower socioeconomic status (SES). However, a higher average personal annual income, which was considered an indicator for a higher SES, was also found in neighborhoods with lower coverage of BCSP. For these neighborhoods, that have a relatively low and high SES, the negative association between the percentage of foreign residents, average household size, and average personal annual income and the coverage in the BCSP had the smallest regression coefficient and 95% confidence interval (CI) values were −0.75 (95% CI: −0.85, −0.65), −13.59 (95% CI: −15.81, −11.37), and −1.05 (95% CI: −1.18, −0.92), respectively, for the 10th quantile. The neighborhoods with higher coverage of opportunistic screening had a relatively higher average personal annual income, with the largest regression coefficient of 1.72 (95% CI: 1.59, 1.85) for the 90th quantile.

**Conclusions:** Women from relatively low and high SES neighborhoods tend to participate less in the BCSP, whereas women with a relatively high SES tend to participate more in opportunistic screening. For women from low SES neighborhoods, tailored interventions are needed to improve the coverage of BCSP.

## Introduction

Worldwide, breast cancer is the most common cause of cancer death in women (1). In 2018 the global age-standardized incidence and mortality rates of breast cancer were 54.4 and 11.6 per 100,000 women, respectively (1). Randomized controlled trials have confirmed that mammography screening can reduce the risk of breast cancer mortality by 20% for women aged 50–70 who attend this screening (2). The European guideline for quality assurance in breast cancer screening and diagnosis suggests to strive for 70% screening coverage in order to have a significant effect on breast cancer burden in the population. However, this percentage is not obtained in many countries where a breast cancer screening program (BCSP) has been established. In Europe, the mean screening coverage is about 50% (range 28–92%), meaning that a large proportion of women who are eligible for screening are not covered by the population breast cancer screening programs. This does not mean that these women do not receive any form of BC screening, since opportunistic BC screening exists in many countries (3).

To understand the reasons for this low coverage, several studies assessed the determinants of screening coverage. The main reported determinants of low screening coverage were related to low socioeconomic status (SES): lower income level, having an immigrant background, and being a single parent (4–7). The conclusions of these studies are mainly derived from linear regression modeling, in which the change in the mean coverage is estimated as a function of the explanatory variables. However, the determinants of the lowest and highest screening coverage are more of interest to policymakers than the determinants of the average coverage. In order to improve the screening coverage of BCSP, it is important to know the characteristics of the women with the lowest coverage in the BCSP, as well as those of the women with the highest coverage in the opportunistic screening. These insights can assist policy makers to implement specific actions to increase the screening coverage of BCSP, especially for those groups that are not covered by opportunistic screening either.

The aim of this study was therefore to evaluate the effect of socio-demographic factors and indicators for SES at the lower end of screening coverage in the BCSP and the higher end of opportunistic screening coverage. Data of screening coverage in Flanders, Belgium were analyzed with a quantile regression model (8).

## Methods

### Screening in Flanders

Flanders, the largest and most populated region in Belgium (around 6 million inhabitants), is among the regions with the highest BC incidence in Europe, despite the early implementation of a BCSP in 2001, which offers biennial screening to women between 50 and 69 years (1, 9). Women in Flanders can also choose to be screened for BC in opportunistic screening outside the BCSP. Where the BCSP is fully reimbursed by health insurance, opportunistic screening does not have a systematic quality control such as daily quality checks of mammography equipment, double reading and case-based feedback to readers (10). Although these two screening strategies coexist in Flanders, the combined coverage rate is only 64.1%, with a coverage rate of 50.0% for the BCSP and 14.1% for the opportunistic screening (11).

### Data Source

For this analysis, publicly available data of 2017 from the Center for Cancer Detection and regional authorities in Flanders were linked at a statistical sector level. The statistical sector level is the smallest level at which administrative information is systematically collected in Flanders and is comparable to the neighborhood in literature (12). We will therefore use the term neighborhood hereafter. From the Center for Cancer Detection data were received of the screening invitations by the BCSP and attendances at the BCSP. The attendances to the opportunistic screening were identified from reimbursement data of the InterMutualist Agency (IMA). The data of opportunistic screening was sent to the Center for Cancer Detection regularly. These data were collected in a collaboration between the Belgian Cancer Registry and the Center for Cancer Detection, as described by their respective statutes (13, 14). From the regional authorities data on socio-demographic characteristics were retrieved (15). Linkage was performed in a protected environment and provided at an aggregated neighborhood level to mask all the individual level information. To prevent identification of any individual, neighborhoods were excluded with less than five women screened. Due to that, 8,690 of the 9,490 neighborhoods in Flanders that provided the data of screening coverage rate in the BCSP and the opportunistic screening were included in this study. For these reasons, ethical approval was not needed for this study because the above-noted measures were taken for privacy protection.

### Study Variables

The primary outcome in this analysis was the coverage rate of screening in the BCSP, which was defined as the percentage of women aged 50–69 screened in the BCSP. The secondary outcome in this analysis was the coverage rate of opportunistic screening which was defined as the percentage of women aged 50–69 screened in the opportunistic screening.

The socio-demographic variables per neighborhood which can characterize the study population from different angles were used as covariates are listed in Table 1 and were defined as follows: (1) *Population density:* the number of residents per km^2^; (2) *Same address as last year:* the percentage of residents with the same address as last year; (3) *Single parents:* the percentage of unmarried residents that are single and live together with at least one child; (4) *Married resident with child(ren) living at home*: the percentage of married residents that live together with at least one child; (5) *Unmarried cohabiting resident with child(ren) living at home:* the percentage of unmarried residents that live together with at least one child; (6) *The percentage of foreign residents:* the percentage of residents without Belgian nationality; (7) *Average personal annual income:* the quotient of the total net taxable income and number of residents on January 1 of the tax year; (8) *Average household size:* the average number of persons in a household.

**Table 1:** Description of the neighborhood-level socio-demographic variables and the coverage in the BCSP at the 10th (Q10) to the 90th (Q90) quantiles^*^.

**Variables**	**Q10**	**Q20**	**Q30**	**Q40**	**Q50**	**Q60**	**Q70**	**Q80**	**Q90**
**Outcome**									
Coverage in the BCSP (%)	33.5	40.0	43.8	47.2	50.0	53.2	56.2	59.9	64.5
**Determinants**									
Population density (1,000 residents per km^2^)	1.1	1.4	1.4	1.4	1.1	1.3	1.1	1.1	0.9
Same address as last year (%)	91.7	91.9	92.3	92.5	93.1	93.1	93.6	93.6	94.3
Single parent (%)	3.8	3.5	3.4	3.3	3.3	3.2	3.1	3.1	3.0
Married resident with child(ren) living at home (%)	20.5	19.7	19.7	20.2	20.7	20.4	20.9	21.3	21.9
Unmarried cohabiting resident with child(ren) living at home (%)	6.4	6.2	6.4	6.2	6.3	6.2	6.3	6.1	6.1
The percentage of foreign residents (%)	11.1	6.2	4.8	4.4	3.9	3.7	3.7	3.4	3.9
Average household size (number)	2.5	2.4	2.4	2.4	2.4	2.4	2.4	2.5	2.5
Average personal annual income (1,000€)	21.1	20.1	20.0	20.1	19.9	19.9	19.8	19.9	19.5

* *The neighborhoods were ranked based on the coverage in the BCSP. The numbers in the table reflect the median value of the determinants per quantile of the coverage in the BCSP*.

### Statistical Analysis

Women were categorized into two groups, the group that was screened in the BCSP and the group that was screened in the opportunistic screening. In the first step of the analysis, the included neighborhoods in the analysis were ranked based on the coverage rates in the BCSP and in the opportunistic screening, respectively, and categorized in 9 quantiles (Q10–Q90). Then the socio-demographic variables were described per quantile. As we have aggregated data, these descriptions reflect the median estimates per quantile.

For each socio-demographic variable, to evaluate the deviations per quantile from the median regression, which is an indicator for the central tendency of the data, multivariable quantile regression models were performed. The quantile regression does not make any assumptions on the distribution of the observations neither on the residuals of the regression model (Supplementary Material Figure 1). The significance of the quantile regression coefficients was tested first to determine if the coefficients were significantly different from 0, then they were tested to determine if the coefficients were significantly different from the median regression coefficient. The quantile regression was considered to provide more information if both tests were significant (8, 16). All statistical tests were two-sided and considered statistically significant at 0.05. All analyses were performed in R 4.0.2.

## Results

### Description of the Coverage and Neighborhoods

Of the 8,690 included neighborhoods in the analysis, the median coverage in the BCSP was 50%. The coverage in the 10th quantile (Q10) was 33.5% and in the 90th quantile (Q90), it was 64.5%. The median percentage of single-parents, the median percentage of foreign residents, and the median average personal annual income decreased with increasing quantiles of BCSP coverage: for Q10–Q90 of the BCSP coverage, the median per quantile decreased from 3.8 to 3.0%, 11.1 to 3.9%, and €21,100 to 19,500, respectively (Table 1). The median coverage of the opportunistic screening was 12%, and for the Q10–Q90 this increased from 4.2 to 24.8%. The median average personal annual income increased with increasing quantiles of the coverage of opportunistic screening from €18,700 to 21,700 for the Q10 to Q90 (Table 2).

**Table 2:** Description of the neighborhood-level socio-demographic variables and coverage of the opportunistic screening at the 10th (Q10) to the 90th (Q90) quantiles^*^.

**Variables**	**Q10**	**Q20**	**Q30**	**Q40**	**Q50**	**Q60**	**Q70**	**Q80**	**Q90**
**Outcome**									
Coverage of the opportunistic screening (%)	4.2	6.6	8.3	10.1	12.0	13.9	16.5	19.4	24.8
**Determinants**									
Population density (1,000 residents per km^2^)	0.8	1.2	1.1	1.3	1.3	1.3	1.2	1.2	1.1
Same address as last year (%)	94.1	93.5	93.1	93.3	93.2	92.9	93.0	92.8	93.2
Single parent (%)	3.1	3.2	3.2	3.3	3.2	3.3	3.3	3.3	3.2
Married resident with child(ren) living at home (%)	21.6	20.7	21.2	20.6	20.7	20.4	20.3	20.3	21.1
Unmarried cohabiting resident with child(ren) living at home (%)	5.8	5.9	5.9	6.0	6.1	6.3	6.4	6.4	6.4
The percentage of foreign residents (%)	4.2	4.5	4.4	3.8	4.4	4.2	3.7	4.2	4.4
Average household size (number)	2.5	2.4	2.4	2.4	2.4	2.4	2.4	2.4	2.5
Average personal annual income (1,000€)	18.7	18.8	19.1	19.3	19.7	19.8	20.5	20.6	21.7

* *The neighborhoods were ranked based on the coverage of the opportunistic screening. The numbers in the table reflect the median value of the determinants per quantile of the coverage of the opportunistic screening*.

### Determinants of the Coverage in the BCSP

**Figure 1:**
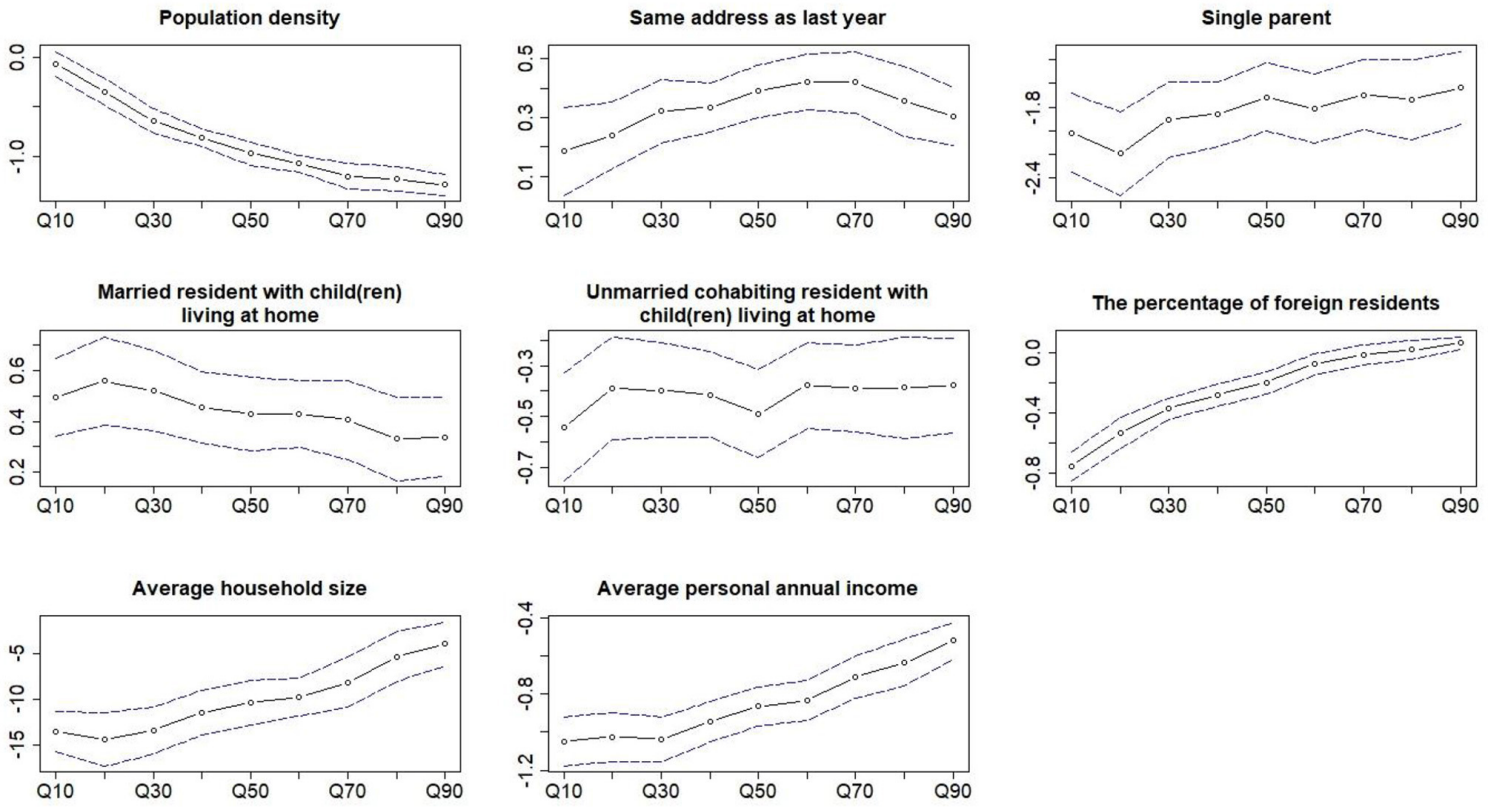
Coefficients of the multivariable quantile regression of all the covariates as a function of the different quantiles of the coverage in the BCSP. The dotted line and the blue dash lines are the coefficient and the 95% CI of quantile regression at the different quantiles of the outcome.

A significant difference between the coefficients of the quantile regression and the median regression was observed for the population density, the percentage of foreign residents, the average household size, and the average personal annual income (Figure 1) (Supplementary Material Figure 2). These four determinants were all negatively associated with the coverage of BCSP with median regression coefficients of −0.97 (95% CI: −1.09, −0.85), −0.20 (95% CI: −0.27, −0.13), −10.38 (95% CI: −12.84, −7.92), and −0.86 (95% CI: −0.97, −0.76), respectively (Table 3). At Q10, the statistically significant association of the quantile regression of the percentage of foreign residents, average household size, and average personal annual income was stronger than the median regression, and the difference was statistically significant (Table 3). Of these three determinants, the regression coefficients and 95% CI for the Q10 of the coverage were −0.75 (95% CI: −0.85, −0.65), −13.59 (95% CI: −15.81, −11.37), and −1.05 (95% CI: −1.18, −0.92), respectively.

**Table 3:** Multivariable quantile regression coefficient and 95%CI of the determinants of coverage in the BCSP at the 10th (Q10) to the 90th (Q90) quantiles.

**Variables**	**Q10**	**Q20**	**Q30**	**Q40**	**Q50**	**Q60**	**Q70**	**Q80**	**Q90**
Population density (1,000 residents per km^2^)	−0.07	**−0.35**	**−0.65**	**−0.81**	**−0.97**	**−1.07**	**−1.20**	**−1.23**	**−1.29**
	(−0.19, 0.06) 77.02^***^	(−0.49, −0.21) 38.95^***^	(−0.77, −0.52) 20.18^***^	(−0.90, −0.72) 7.80^**^	(−1.09, −0.85)	(−1.16, −0.99) 3.67	(−1.32, −1.07) 19.09^***^	(−1.35, −1.11) 23.68^***^	(−1.39, −1.18) 14.72^***^
Same address as last year (%)	**0.19**	**0.24**	**0.32**	**0.34**	**0.39**	**0.42**	**0.42**	**0.36**	**0.30**
	(0.04, 0.34) 5.82^*^	(0.13, 0.35) 4.32^*^	(0.21, 0.43) 1.48	(0.25, 0.42) 0.85	(0.30, 0.48)	(0.32, 0.52) 0.83	(0.32, 0.53) 0.22	(0.23, 0.48) 0.29	(0.21, 0.40) 0.99
Single parent (%)	**−2.02**	**−2.19**	**−1.91**	**−1.86**	**−1.72**	**−1.81**	**−1.70**	**−1.74**	**−1.64**
	(−2.36, −1.68) 1.38	(−2.55, −1.84) 5.77^*^	(−2.23, −1.58) 1.77	(−2.13, −1.58) 1.24	(−0.21, −1.42)	(−2.11, −1.52) 0.97	(−1.99, −1.40) 0.01	(−2.08, −1.40) 0.01	(−1.95, −1.33) 0.19
Married resident with child(ren) living at home (%)	**0.49**	**0.56**	**0.52**	**0.45**	**0.43**	**0.43**	**0.41**	**0.33**	**0.34**
	(0.34, 0.65) 0.41	(0.38, 0.73) 1.54	(0.36, 0.68) 1.67	(0.31, 0.59) 0.19	(0.29, 0.57)	(0.30, 0.56) 0.00	(0.25, 0.56) 0.14	(0.17, 0.50) 1.13	(0.18, 0.49) 0.93
Unmarried cohabiting resident with child(ren) living at home (%)	**−0.54**	**−0.39**	**−0.40**	**−0.41**	**−0.49**	**−0.38**	**−0.39**	**−0.39**	**−0.38**
	(−0.75, −0.33) 0.23	(−0.59, −0.19) 1.28	(−0.58, −0.21) 1.85	(−0.58, −0.25) 1.16	(−0.66, −0.32)	(−0.55, −0.21) 3.79	(−0.56, −0.22) 1.36	(−0.59, −0.19) 0.86	(−0.57, −0.19) 0.78
The percentage of foreign residents (%)	**−0.75**	**−0.53**	**−0.37**	**−0.28**	**−0.2**	**−0.08**	−0.02	0.01	**0.06**
	(−0.85, −0.65) 76.47^***^	(−0.64, −0.43) 28.66^***^	(−0.44, −0.30) 44.30^***^	(−0.35, −0.21) 8.66^**^	(−0.27, −0.13)	(−0.15, −0.01) 31.97^***^	(−0.08, 0.05) 41.31^***^	(−0.05, 0.08) 39.35^***^	(0.02, 0.10) 50.62^***^
Average household size (number)	**−13.59**	**−14.41**	**−13.39**	**−11.51**	**−10.38**	**−9.78**	**−8.20**	**−5.36**	**−4.01**
	(−15.81, −11.37) 3.18^*^	(−17.31, −11.50) 6.68^**^	(−15.95, −10.84) 8.73^**^	(−13.95, −9.07) 2.33	(−12.84, −7.92)	(−11.87, −7.69) 0.34	(−10.92, −5.48) 3.38^*^	(−8.09, −2.63) 9.47^**^	(−6.41, −1.60) 12.53^***^
Average personal annual income (1,000€)	**−1.05**	**−1.02**	**−1.04**	**−0.95**	**−0.86**	**−0.83**	**−0.71**	**−0.63**	**−0.52**
	(−1.18, −0.92) 4.90^*^	(−1.15, −0.89) 4.46^*^	(−1.15, −0.92) 9.34^**^	(−1.05, −0.84) 5.02^*^	(−0.97, −0.76)	(−0.94, −0.73) 0.85	(−0.82, −0.60) 20.28^***^	(−0.76, −0.51) 18.82^***^	(−0.62, −0.42) 44.61^***^

*Numbers in bold: The 10th (Q10) to the 90th (Q90) quantile regression coefficient that was significantly different from zero at the 5% significance level*.

*The 95%CI in parentheses. F statistics below the parentheses*.

* *P < 0.05*,

** *P < 0.01*,

*** *P < 0.001*.

### Determinants of Coverage of the Opportunistic Screening

**Figure 2:**
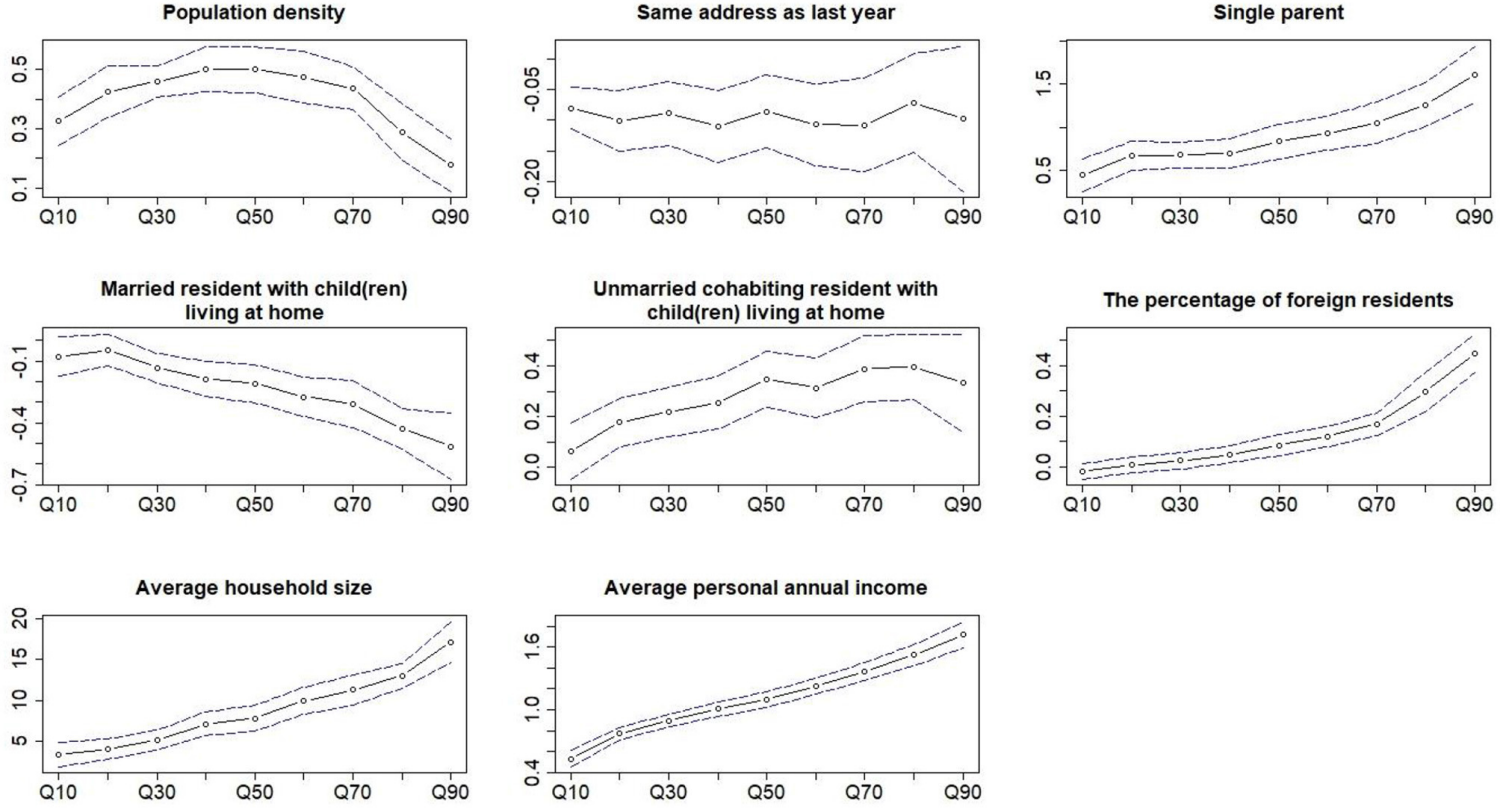
Coefficients of the multivariable quantile regression of all the covariates as a function of the different quantiles of the coverage of the opportunistic screening. The dotted line and the blue dash lines are the coefficient and the 95% CI of quantile regression at the different quantiles of the outcome.

The coefficients of the quantile regression for most of the determinants were significantly different from the median regression (Figure 2) (Supplementary Material Figure 3). The percentage of single parent, average household size, and average personal annual income were positively associated with the coverage of opportunistic screening. For these three variables, the coefficients and 95% CI of the quantile regression for the Q90 were 1.61 (95% CI: 1.28, 1.94), 17.15 (95% CI: 14.66, 19.63), and 1.72 (95% CI: 1.59, 1.85), respectively, which was significantly larger than the median regression coefficients 0.83 (95% CI: 0.64, 1.03), 7.86 (95% CI: 6.34, 9.38), and 1.10 (95% CI: 1.02, 1.17), respectively (Table 4).

**Table 4:** Multivariable quantile regression coefficient and 95%CI of the determinants of coverage of the opportunistic screening at the 10th (Q10) to the 90th (Q90) quantiles.

**Variables**	**Q10**	**Q20**	**Q30**	**Q40**	**Q50**	**Q60**	**Q70**	**Q80**	**Q90**
Population density (1,000 residents per km^2^)	**0.33**	**0.42**	**0.46**	**0.50**	**0.05**	**0.47**	**0.44**	**0.29**	**0.18**
	(0.24, 0.41) 9.65^**^	(0.34, 0.51) 2.48	(0.41, 0.51) 1.07	(0.42, 0.58) 0.00	(0.42, 0.58)	(0.39, 0.56) 1.09	(0.36, 0.51) 3.71	(0.20, 0.38) 6.18^*^	(0.09, 0.27) 9.44^**^
Same address as last year (%)	**−0.08**	**−0.10**	**−0.09**	**−0.11**	**−0.09**	**−0.11**	**−0.11**	−0.07	−0.10
	(−0.11, −0.05) 0.01	(−0.15, −0.05) 0.16	(−0.14, −0.04) 0.01	(−0.17, −0.05) 1.19	(−0.15, −0.02)	(−0.17, −0.04) 0.81	(−0.18, −0.03) 0.44	(−0.15, 0.01) 0.04	(−0.22, 0.02) 0.04
Single parent (%)	**0.45**	**0.67**	**0.68**	**0.7**	**0.83**	**0.93**	**1.05**	**1.26**	**1.61**
	(0.26, 0.64) 6.56^*^	(0.50, 0.84) 1.93	(0.52, 0.83) 3.88^*^	(0.52, 0.87) 4.01^*^	(0.64, 1.03)	(0.73, 1.12) 1.61	(0.81, 1.30) 2.81	(1.01, 1.51) 8.66^**^	(1.28, 1.94) 17.84^***^
Married resident with child(ren) living at home (%)	−0.08	−0.05	**−0.13**	**−0.19**	**−0.21**	**−0.27**	**−0.31**	**−0.43**	**−0.51**
	(−0.17, 0.02) 5.32^*^	(−0.12, 0.03) 12.45^***^	(−0.20, −0.06) 5.81^*^	(−0.27, −0.10) 0.40	(−0.30, −0.12)	(−0.37, −0.18) 6.25^*^	(−0.42, −0.91) 8.20^**^	(−0.53, −0.33) 15.34^***^	(−0.67, −0.36) 19.06^***^
Unmarried cohabiting resident with child(ren) living at home (%)	0.06	**0.18**	**0.22**	**0.26**	**0.35**	**0.31**	**0.39**	**0.40**	**0.33**
	(−0.05, 0.17) 19.61^***^	(0.08, 0.27) 13.68^***^	(0.12, 0.32) 6.40^*^	(0.15, 0.36) 4.60^*^	(0.24, 0.46)	(0.20, 0.43) 1.02	(0.26, 0.52) 0.67	(0.27, 0.52) 0.30	(0.14, 0.53) 0.02
The percentage of foreign residents (%)	−0.02	0.01	0.02	**0.05**	**0.08**	**0.12**	**0.17**	**0.30**	**0.45**
	(−0.05, 0.01) 20.10^***^	(−0.02, 0.04) 11.57^***^	(−0.01, 0.06) 12.26^***^	(0.02, 0.08) 10.31^**^	(0.04, 0.13)	(0.08, 0.16) 11.67^***^	(0.12, 0.21) 18.69^***^	(0.22, 0.37) 28.05^***^	(0.37, 0.52) 146.57^***^
Average household size (number)	**3.45**	**4.05**	**5.23**	**7.17**	**7.86**	**9.96**	**11.30**	**13.05**	**17.15**
	(1.95, 4.94) 37.66^***^	(2.82, 5.27) 42.37^***^	(3.97, 6.49) 21.00^***^	(5.71, 8.63) 1.43	(6.34, 9.38)	(8.32, 11.60) 16.69^***^	(9.41, 13.19) 26.22^***^	(11.52, 14.57) 34.50^***^	(14.66, 19.63) 83.72^***^
Average personal annual income (1,000€)	**0.53**	**0.77**	**0.90**	**1.01**	**1.10**	**1.23**	**1.36**	**1.53**	**1.72**
	(0.45, 0.61) 227.80^***^	(0.70, 0.83) 71.65^***^	(0.84, 0.96) 40.08^***^	(0.94, 1.08) 11.31^***^	(1.02, 1.17)	(1.15, 1.30) 26.09^***^	(1.28, 1.45) 47.88^***^	(1.42, 1.63) 63.88^***^	(1.59, 1.85) 49.11^***^

*Numbers in bold: The 10th (Q10) to the 90th (Q90) quantile regression coefficient that was significantly different from zero at the 5% significance level*.

*The 95%CI in parentheses. F statistics below the parentheses*.

* *P < 0.05*,

** *P < 0.01*,

*** *P < 0.001*.

## Discussion

We found that a larger percentage of foreign residents and a larger average household size as indicators for lower SES were found in neighborhoods with a lower coverage in the BCSP. However, a higher average personal annual income as an indicator for a higher SES was also found in neighborhoods with a lower coverage in the BCSP. For these neighborhoods, the negative association between SES and coverage in the BCSP was stronger than in the neighborhoods with a relatively middle SES level. The neighborhoods with a higher coverage of opportunistic screening had a relatively higher average personal annual income and the positive association between SES and coverage of the opportunistic screening was stronger than in neighborhoods with a relatively lower SES level.

The median coverage in the BCSP for all neighborhoods was 50%. The median coverage for neighborhoods in the lowest and the highest quantile was 33.5 and 64.5%, respectively. The coverage in the BCSP in Flanders was lower than in countries like the Netherlands and the United Kingdom where BC screening is mainly performed in the BCSP, but it was close to countries like France and Germany where both BCSP and opportunistic screening are performed for BC screening (17). As the median coverage of the opportunistic screening was 12%, with 4.2% coverage in the neighborhoods in the lowest quantile and 24.8% coverage in the neighborhoods in the highest quantile, the combined coverage in the BCSP and the opportunistic screening was relatively close to the BC screening coverage in other Western European countries (3, 17).

We observed that women in neighborhoods with a lower SES, as indicated by a crowded housing condition (18, 19) or being an immigrant with a foreign nationality (20), tended to participate less in the BCSP than women in neighborhoods with middle level SES. The negative association between these variables and the coverage in the BCSP was previously also found in other European countries (21, 22). An explanation for this phenomenon is that women with a relatively low SES have a possible lack of health literacy, leaving them less informed on the benefit of BCSP (23). Other reasons may include language barriers for women with an immigration background (24), and the fact that the hardship of life for women with relatively low SES may require more time to work than high SES women and reduce the attention to health care (25).

Interestingly, we also found that women in neighborhoods with a higher income as indicated by a higher average personal annual income also tend to participate less in the BCSP than the women in neighborhoods with middle level income. However, these women tend to participate more in the opportunistic screening than the women in neighborhoods with middle level income. Studies in France, where BCSP and opportunistic screening coexist as in Flanders, also found that women in the most affluent group tend to participate more in opportunistic screening (26). A possible explanation for this phenomenon is that women who belong to the neighborhoods with a relatively high SES may prefer the opportunistic screening which has a more flexible time schedule and more personalized service than the BCSP (2, 27).

## Strength and Limitation

The strength of this study is that the validity of the screening coverage data was warranted by the administrative database of the screening program in Flanders. The recall bias in self-reported surveys was avoided in our data (28). Moreover, the determinants of screening coverage were evaluated for the full distribution of the coverage in the BCSP and the opportunistic screening. The heterogeneous effect of the determinants of screening coverage in different quantiles can provide a more specific target for the potential interventions to improve the screening coverage rate. A limitation of this study was that all data were aggregated at a neighborhood level. It was therefore not possible to explore the variation within the neighborhoods due to the aggregated nature of the data. Moreover, the aggregated data in the current data do not contain factors that have a potential impact on the implementation of the screening, such as women's contract information with general practitioners which might play an important role in the implementation of opportunistic screening. However, studies have shown that residents who live in the same neighborhood share similar SES and health behavior (29, 30) and the association between the determinants in our study and the screening coverage was consistent with the literature. Therefore, the findings in this study can demonstrate the disproportional impact of SES on women who have different breast cancer screening rate and can inform the resource allocation in policymaking. Another potential limitation of this study was the exclusion of neighborhoods that have less than five screened women, which was mainly related to the small size of a neighborhood. This measure was taken by the breast cancer screening program administrators with the aim to protect privacy and is mandatory by the Flemish government. However, we think that the effect of this exclusion was limited due to the fact that we included 92% of all neighborhoods (31).

## Conclusion

Women in neighborhoods that have a relatively low SES, that are characterized by being an immigrant with a foreign nationality, and having a large average household size, as well as women in neighborhoods that have a relatively high SES, that are characterized by a high average personal annual income, participate less frequently in the BCSP. On the other hand, women who belong to neighborhoods with a relatively high SES tend to participate more in the opportunistic screening. Tailored intervention that aims to increase the coverage of BCSP should pay more attention to women in these neighborhoods.

## Data Availability Statement

The original contributions presented in the study are included in the article/Supplementary Material, further inquiries can be directed to the corresponding author/s.

## Ethics Statement

Ethical review and approval was not required for the study on human participants in accordance with the local legislation and institutional requirements. Written informed consent for participation was not required for this study in accordance with the national legislation and the institutional requirements.

## Author Contributions

LD, MJG, GB, and GV conceived the study, performed the data analysis, and wrote the manuscript. SJ was involved in the data curation and review of the manuscript. KV, MG, HD, and PM wrote the manuscript and verified the analysis. All authors discussed and interpreted the results and contributed to the final manuscript.

## Conflict of Interest

The authors declare that the research was conducted in the absence of any commercial or financial relationships that could be construed as a potential conflict of interest.
